# The Transverse Process as a Landmark for Estimating Dural Sac Depth and Feasible Planes for Optimized Paramedian Needle Insertions †

**DOI:** 10.3390/neurosci6040119

**Published:** 2025-11-21

**Authors:** Anna Puigdellívol-Sánchez, Hipólito Labandeyra, Alberto Prats-Galino, Xavier Sala-Blanch

**Affiliations:** 1 Laboratory of Surgical Neuroanatomy (LSNA), Human Anatomy and Embryology Unit, Faculty of Medicine and Health Sciences, Universitat de Barcelona, 08036 Barcelona, Spain; 2 Medicina de Família, CAP Anton de Borja-Centre Universitari, Consorci Sanitari de Terrassa, 08191 Rubí, Spain; 3 Division of Regional Anesthesia, Anesthesiology Service, HM Delfos Hospital, 08023 Barcelona, Spain; 4 Division of Regional Anesthesia, Anesthesiology Service, Hospital Clínic de Barcelona, 08036 Barcelona, Spain

**Keywords:** regional anesthesia, angles, ex vivo, transverse process, spinal needle, needle insertion, spinal anesthesia, lumbar vertebrae, anatomical landmarks, dural sac area

## Abstract

Background: The skin-to-transverse process distance (st) correlates with the skin-to-dural sac depth (d) and may be used to estimate optimal angles for perpendicular needle insertion using the formula inverse cosine d/√(1 + d^2^), as outlined in free visual guides. Objective: We aimed to analyze the relationship between the transverse process and dural sac depth at lumbar levels relevant to spinal anesthesia and to determine the range of planes where perpendicular paramedian needle insertion is feasible when midline access is not viable. Methods: Ten ex vivo trunks were flexed using an abdominal support, and CT scans were performed. Correlations between the transverse process and dural sac depth were evaluated from L3 to S1. Perpendicular planes at the level of needle paths were examined at L3–L4 and L4–L5. Median path viability was assessed. Results: The transverse process aligned with the dorsal dural sac at L3, the posterior third at L4, and the middle zone at L5 or S1. Median needle insertion was not viable in 20–30% of L4–L5 and L3–L4 levels, respectively. However, paramedian access was possible. The vertical range of viable paramedian planes was 8.7 ± 2.9 mm (L4–L5) and 7.9 ± 1.9 mm (L3–L4). Coronal reconstructions showed that the upper level of the transverse process correlates with the skin-perpendicular planes where insertion is likely to succeed. Conclusion: Many elderly spines lack viable midline paths. The superior aspect of the transverse process serves as a useful landmark for estimating dural sac depth, calculating paramedian angles, and identifying the plane for successful perpendicular needle insertion.

## 1. Introduction

The ultrasound visualization of only the posterior complex of the dura mater is a predictor of difficult spinal anesthesia [1]. A progressive reduction in the interlaminar space occurs with age [2] or due to osteoporotic vertebral body compression fractures [3]. Furthermore, the interspinous ligament may often be calcified [4,5]. For these reasons, the median approach may sometimes be impeded [3,4,5], requiring a paramedian approach instead [6,7,8].

**Figure 1:**
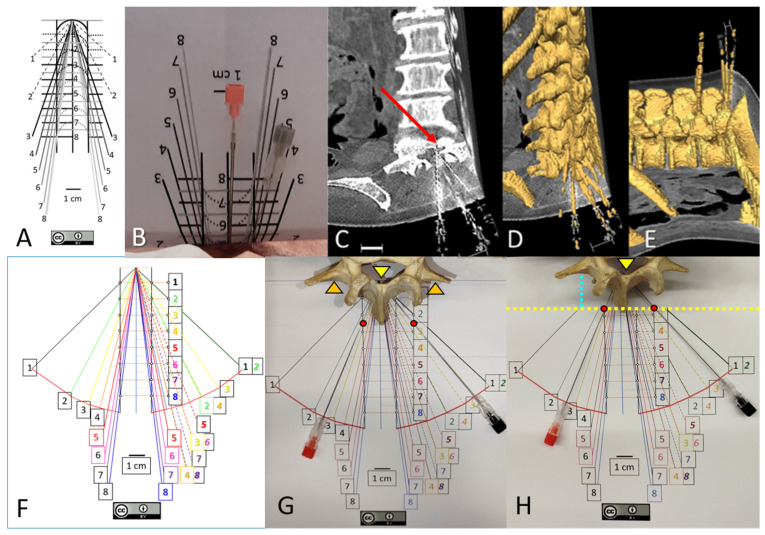
(**A**–**E**) reproduced from [3], under a CC-BY license. (**A**) Original free visual guide for 1 cm paramedian approaches [10]. (**B**) After measuring the skin–dural sac distance (d) by ultrasound (5 cm in this specific case), the guide must be folded along the horizontal line corresponding to that distance, while the needle should be inserted 1 cm paramedian, perpendicular to the skin, by following the corresponding oblique line. (**C**). Computed tomography of the specimen. Axial and coronal planes are shown. Needles were introduced medially and paramedially at 1 cm on the right side, and at individualized paramedian distances on the left side. The red arrow indicates how the left needle reaches the most ventral part of the spinal canal and slightly penetrates the vertebral body. (**D**) 3D reconstruction. E. Prone position model, illustrating successful perpendicular needle insertions. (**F**) Modified version of the original guide with color changes for better visualization of the needle angle according to the measured (d). The modified guide may be downloaded from the Supplementary Material and must be printed at 1:1 scale. Insertion angles for needle insertions at 2 cm paramedian, where the best interlaminar window is usually observed [3], are shown here in italics. Note that insertion angles at 2 cm paramedian for (d) values of 2, 4, 6, and 8 cm correspond to the insertion angles at 1 cm paramedian for (d) values of 1, 2, 3, and 4 cm, respectively. A red curved line is marked at 90 mm from the target, coinciding with the maximal length of most commonly used spinal needles, where penetration should be stopped. (**G**) Simulation of a needle insertion following the guide. Note that in this real vertebra, the transverse process (orange arrowheads) is aligned with the dorsal part of the spinal canal, where the needle tips are located (yellow arrowhead). (**H**) The blue discontinuous line corresponds to the skin-to-transverse process distance, while the tip of the spinous process may correspond to the skin level (2.5 cm in this specific case). Therefore, the guide is folded at 2.5 cm. The insertion of the spinal needles at the 1 cm paramedian (**left**) and 2 cm paramedian (**right**) positions -indicated by red circles- was stopped upon reaching the red curved line.

The optimal angles for paramedian approaches depend on the skin-to-dural sac distance (d) and are defined as ‘arccos [d/√(1 + d^2^)]’, but the maximal and minimal paramedian angles for reaching the spinal canal and dural sac differ by less than 5° from the optimal angles [9]. To improve needle orientation, a visual guide (Figure 1A) was constructed using the measurement tools in Microsoft PowerPoint. It consisted of parallel paramedian vertical lines drawn 1–2 cm from the midline, and horizontal lines at 1 cm intervals from the target. Oblique lines, representing the intended needle paths, connected each potential paramedian entry point at different distances (d) to the target. This guide is freely available for download from the University of Barcelona’s public repository at https://diposit.ub.edu/dspace/handle/2445/179594 [accessed on 10 November 2025] [10], An enhanced color version (Figure 1F), featuring a red visual curve indicating a point 9 cm from the dural sac (the typical length of a spinal needle), is available in the Supplementary Material of this manuscript and must be printed at a 1:1 scale. A high rate of successful experimental needle insertions can be achieved by using the visual guide for paramedian angles, based on the skin-to-dural sac distance [3,10].

Clinical experience with pre-procedural ultrasound imaging [8] to locate the ideal space for spinal needle placement [11] or to pre-estimate insertion angles [12] has significantly reduced the number of needle insertion attempts. However, interspinous ligament calcification [4,5] may interfere with the estimation of the skin-to-dural sac distance used to calculate the optimal insertion angle. To address this limitation, we recently described a strong mathematical correlation between the skin-to-dural sac distance (d) and the skin-to-transverse process distance (st) [3]. Although perpendicular needle insertions are feasible in preserved spines [3,6], the visual guide also provides a reproducible needle insertion angle for paramedian approaches, but accidental penetration in osteoporotic bones might be confused by the loss of resistance sensation when traversing the flavum ligament. So, the maximal penetration depending on the dural sac depth must be taken into account [3] and the insertion level must be clearly identified.

## 2. Methods

This anatomical study of anesthetic approaches received approval from the Ethics Committee of the University of Barcelona (IRB00003099), with informed consent waived for the use of non-identifiable ex vivo samples and related images. The average age of the ex vivo donors at admission during the study period was 89.1 ± 7.8 years for females and 82.5 ± 11.5 years for males. Permission was granted by the Head of the Donor Service to obtain images of museum specimens located in the Dissection Room of the Faculty of Medicine and Health Sciences.

### 2.1. Ex Vivo Trunks, CT and 3D

Ten randomly selected ex vivo samples, which had been stored at 4 °C without embalming, were examined the day after their arrival at the Donor Service and were placed in the prone position with a support under the abdomen to replicate the lumbosacral spine position during spinal anesthesia. Ultrasound was used to measure either the skin-to-dural sac distance (d) or the skin-to-transverse process distance (st), and needles were inserted perpendicularly to the skin, just below the upper spinous process, using either a median or paramedian approach.

The insertion angle for paramedian approaches was optimized with the aid of a folded visual guide (Figure 1A,B), based on the estimated distance by ultrasound (d). Previously reported data have shown high success rates for these perpendicular needle insertions within the dural sac (Figure 1C–E) compared with blind insertions [3]. In the present study, we describe the anatomical relationship between the transverse process and the dural sac across different intervertebral levels, as well as the range of viable perpendicular trajectories. Following a computed tomography (CT) scan of the lumbosacral region (Figure 1C) using a Somatom Sensation 64 system (Siemens Medical Systems, Erlangen, Germany), Digital Imaging and Communications in Medicine (DICOM) files were analyzed using Amira 5.6 software (Thermo Fisher Scientific, Waltham, MA, USA).

The method for generating 3D models with Amira 5.6 software has been previously described [10]. Briefly, semiautomatic threshold segmentation discriminated voxels corresponding to bone tissue, creating volumes of interest for vertebral structures (Figure 1D,E).

**Figure 2:**
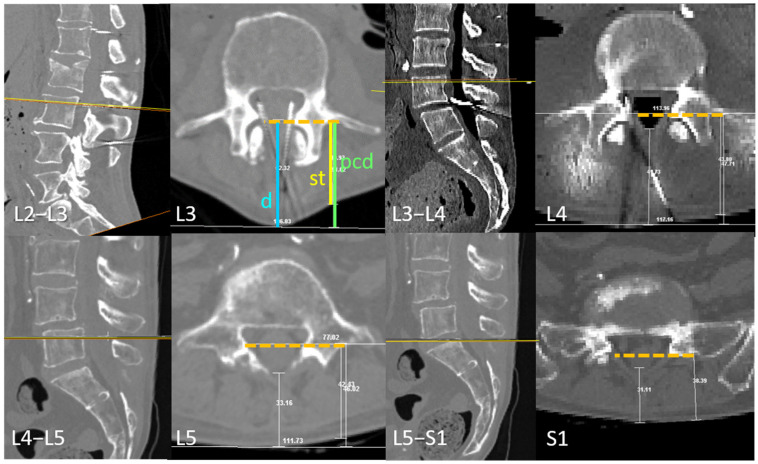
The transverse process and dural sac depth at different intervertebral levels. The illustrated plane is perpendicular to the skin (visualized as an horizontal orange line in the lateral views) and corresponds to the proposed needle insertion trajectory. The skin-to-transverse process distance (st, in yellow) correlates with the skin-to-dural sac distance (d, in blue). This figure highlights cases with the greatest discrepancy between those distances, along with the parallel corrected distance (pcd, in green), representing a theoretical distance to the outermost level of the spinous process in thin patients. Although some cases exhibit a divergence of approximately 1 cm between (d) and either (st) or (pcd), the transverse process depth remains strongly correlated with the maximum intended needle penetration at the most commonly targeted intervertebral levels. The posterior portion of the transverse process, identified via ultrasound, corresponds to the interior of the dural sac at the L4 and L5 vertebrae, precisely where the intrathecal needle is typically placed during attempts at the L3–L4 and L4–L5 intervertebral levels.

Oblique views were adjusted to visualize planes perpendicular to the skin, aligned with the potential trajectory of perpendicular needle insertion (Figure 2).

#### 2.1.1. Transverse Process and Dural Sac Alignment

Planes perpendicular to the skin of the flexed ex vivo specimen, aligned with the first plane in which the transverse process (tp) could be fully visualized, were obtained. These planes were used to describe the anatomical alignment of the transverse process and its correspondence with the depth of the dural sac (ds) (Figure 2).

#### 2.1.2. Medial and Paramedian Paths for Perpendicular Needle Insertions

Sagittal planes were obtained in the flexed trunks to determine whether a median path for needle insertion was feasible at any insertion angle.

**Figure 3:**
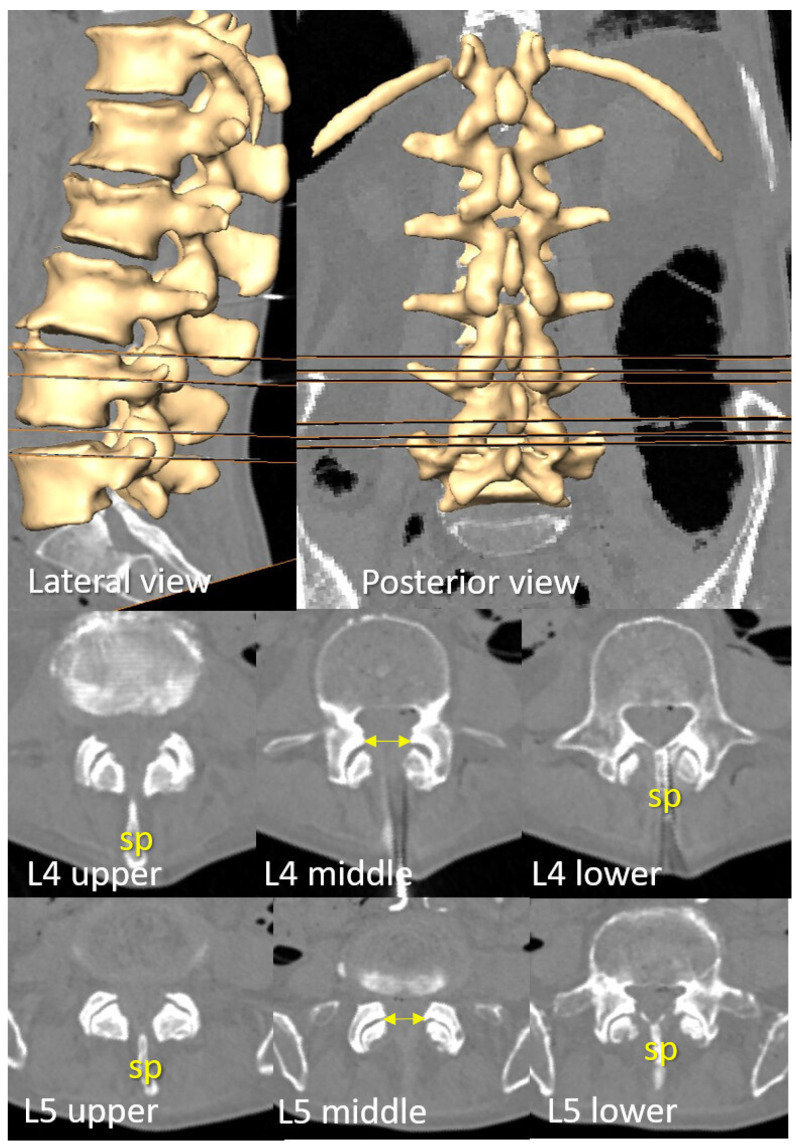
D reconstruction of a representative spine with preserved intervertebral spaces. The upper and lower perpendicular planes to the lumbar skin—where paramedian needle insertion into the dural sac is viable—are displayed in lateral and posterior views of the spine, as well as in axial planes at the L3–L4 and L4–L5 intervertebral levels. Note that the spinous process obstructs median needle insertion at these levels. The level with the widest interlaminar window (indicated by bilateral arrows) is located midway between these planes and coincides with the superior aspect where the (tp) of the L4 and L5 vertebrae become visible, as observable in both axial and coronal views.

The upper and lower axial planes allowing bilateral and paramedian needle insertion at the commonly used intervertebral levels, L3–L4 and L4–L5, were identified, along with the plane presenting the widest interlaminar window. The distance between the upper and lower axial planes was then measured (Figure 3).

**Figure 4:**
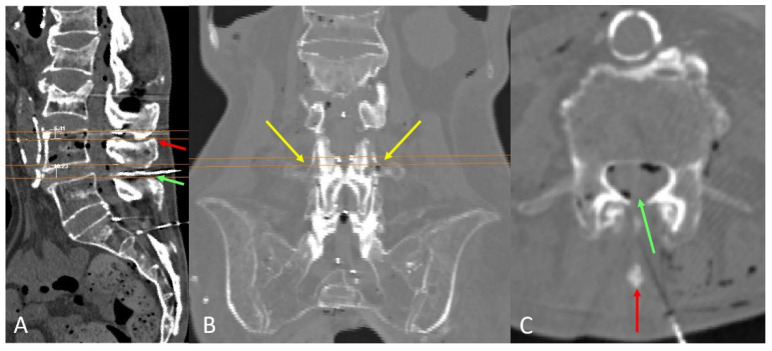
(**A**) A representative case in which the L4–L5 level presents a viable median path (green) for needle insertion, while a prominent spinous process obstructs the midline approach at L3–L4 (red arrow). (**B**) Coronal view of the planes at the level of the transverse process, showing the planes (in orange) suitable for a paramedian and perpendicular approach. This view confirms the alignment of those planes with the upper border of the transverse process (yellow arrows). (**C**) The upper border of the transverse process also indicates the level with a wider interlaminar window, where a paramedian approach is feasible (green arrow), even when the spinous process blocks access via the midline (red arrow).

Coronal planes at the most dorsal and rostral levels of the transverse process were prepared to assess the anatomical correlation between the transverse process and the axial planes with viable paramedian paths (Figure 4).

### 2.2. Museum Models

**Figure 5:**
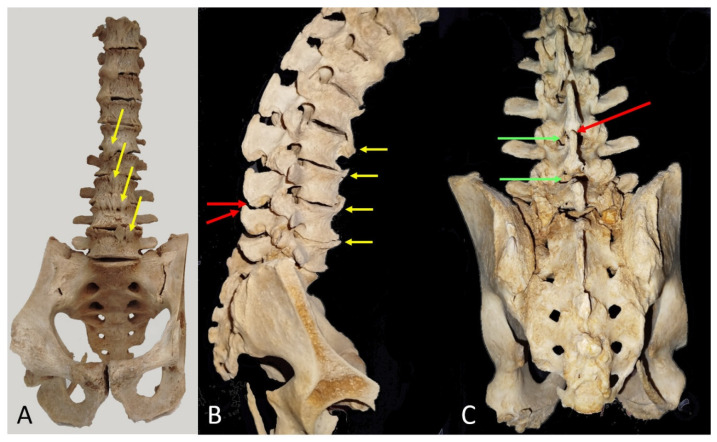
(**A**) An anterior view of a museum model showing anterior syndesmophytes (yellow arrows) at several lumbar levels. (**B**) Lateral view of the same model. The anterior syndesmophytes restrict anterior trunk flexion and consequently limit the widening of the interspinous space, where the spinous process obstructs a potential median needle insertion (red arrows) at the L3–L4 intervertebral level. (**C**) Posterior view. The spinous process obstructing the median L3–L4 approach is also indicated by a red arrow. Green arrows indicate that a perpendicular paramedian approach at the level of the upper part of the spinous process is feasible.

One of the dissected spine models from the Donor Service of the Faculty of Medicine was used. The model was selected for its representation of a spine with degenerative arthrosis, including anterior syndesmophytes -a common condition in elderly patients-. Anterior, sagittal, and posterior views of the model are provided (Figure 5), illustrating the relationship between the (tp) process and the interlaminar window.

**Figure 6:**
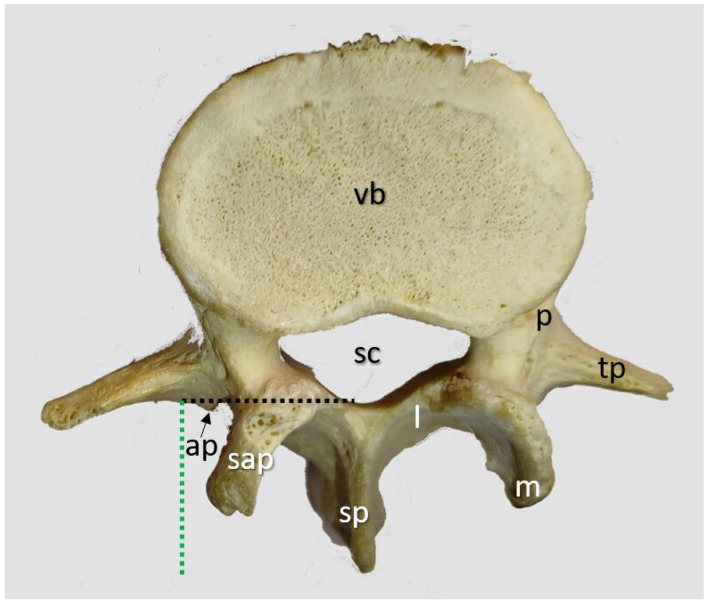
Lumbar L4 vertebra. The transverse process (tp) of the lumbar vertebrae features a small, posteriorly oriented accessory process (ap) medially. The superior articular process (ap) includes a rounded prominence known as the mammillary process (m). The vertebral body (vb), pedicle (p), lamina (l), and spinous process (sp) exhibit a structure consistent with other spinal vertebrae. Notice that the most medial part of the (tp) aligns with the posterior spinal canal (sc) in this case (black dashed lines). Its distance from the spinous process tip, comparable to the skin-to-transverse process distance (st), also serves as an indicator of dural sac depth (green dashed lines).

Additionally, a museum model of a normal vertebra was used to review lumbar vertebral anatomy. A superior view of the L4 vertebra is included (Figure 6) to demonstrate its normal anatomy. The background was removed using Corel PaintShop Pro 2023 Ultimate (Alludo HQ, Ottawa, Canada).

## 3. Results

### 3.1. Transverse Process and Depth Level Within the Dural Sac

The alignment between the dural sac and the dorsal and medial parts of the transverse process varies depending on the vertebral level. In the ex vivo samples studied, this relationship was consistent and followed a rostrocaudal pattern (Figure 2): At the L3 level, the dorsal and medial part of the transverse process aligned with the dorsal part of the dural sac. At L4, it corresponded to the posterior third of the dural sac and at L5 was aligned with the middle zone. In contrast, at S1, this alignment was more variable, ranging from the middle to the posterior zone of the dural sac (Figure 2).

### 3.2. D Reconstruction, Transverse Process and Interlaminar Window

The upper level of the most medial part of the transverse process correlated with the lower plane where an existing interlaminar window allowed needle insertion perpendicular to the skin. The (tp) of the L5 and L4 vertebrae tends to show a certain cranial inclination laterally. The most lateral part of the (tp) is consistently located slightly caudal to the most cranial plane where a needle insertion remains viable. The ideal plane for needle insertion, characterized by a wider interval between articular processes, is positioned slightly cranial to the lower plane (Figure 3).

### 3.3. Viable or Non-Viable Medial Needle Insertions

Table 1 and Appendix A present the quantification of viable intervertebral spaces for medial needle insertion in our case series, along with the measured distances between upper and lower planes allowing a perpendicular (0°) paramedian needle approach relative to the skin surface.

The average distance between the upper and lower planes through which needle insertion was possible was 8.7 ± 2.9 mm at the L4–L5 level and 7.9 ± 1.9 mm at the L3–L4 intervertebral level. In 20% of cases, no viable median path was observed at the L4–L5 level, and in 30% of cases, no viable median path was present at L3–L4.

Figure 4 illustrates a case with a viable median path at the L4–L5 intervertebral level but no viable median path at L3–L4 due to a prominent spinous process (Figure 4A). The viable L3–L4 level where a paramedian approach, perpendicular to the skin, could still be attempted, illustrates the consistent alignment with the upper part of the medial transverse process (Figure 4B,C).

### 3.4. Museum Models

#### 3.4.1. Spine with Anterior Syndesmophytes

A certain degree of arthrosis is present in all ancient patients, where osteophytes forming syndesmophytes are commonly observed, as seen in the selected museum specimen (Figure 5). The anterior syndesmophytes restrict lumbar spine flexion in the patient, preventing the flexed position that would otherwise increase the interspinous distance. Additionally, the enlarged spinous process obstructs median approaches at any angle. In this case, the upper and most lateral part of the transverse process indicates the optimal level for needle insertion perpendicular to the skin, following a pre-estimated paramedian angle based on the distance from the skin to the transverse process.

#### 3.4.2. Lumbar Vertebrae

The normal anatomy of an L4 vertebra is shown in Figure 6.

The most medial part of the (tp) aligns with the posterior aspect of the spinal canal (sc) in this case, and the distance from the posterior part of the (tp) to the tip of the spinous process (almost comparable to the skin level) indicates the depth at which the dural sac will be located.

## 4. Discussion

The high success rate of experimental perpendicular needle insertions when a viable median path is present, along with the mathematical correlation between (d) and (st), has been previously described, where raw data of the specimens may be found [3]. This complementary article describes the detailed anatomical alignment between the transverse process (tp) and the dural sac (ds) per segment, as well as the range of viable perpendicular paths that coincide with the upper part of the transverse process. To our knowledge, this is the first manuscript describing these anatomical relationships in detail, which may help optimize spinal needle insertion.

The high prevalence of older patients in whom a midline approach is not viable [2,4] is comparable to the characteristics of patients admitted to the Donor Service. This supports the proposal of performing a systematic ultrasound examination before attempting needle insertion in the oldest patients, as previously suggested [3,8,11,13]. The presence of anterior osteophytes causing anterior vertebral body bridging (syndesmophytes), which limits lumbar spine flexion, has been reported in 5.3% to 14% of radiographs in patients over 60 years of age, depending on the lumbar level involved [14]. This percentage is likely higher in older age groups and may exceed 65% in patients with diffuse idiopathic skeletal hyperostosis (DISH) [15]. The illustrated case of advanced arthrosis and syndesmophyte formation is likely representative of elderly patients requiring anesthesia for hip fracture repair.

Ultrasound measurement of the (st) distance, when targeting the midzone of the dural sac through the L3–L4 to L5–S1 intervertebral spaces, enhances level identification accuracy, overcoming common palpation errors and compensating for Tuffier’s line variability [16]. Additionally, it will determine the maximum safe needle insertion depth, reducing the risk of vertebral body penetration [3], as previously proposed for psoas compartment block/catheter techniques [17]. Finally, if a median approach is not feasible, ultrasound guidance can help to calculate the optimal angle for a paramedian alternative approach.

The average reduced height of viable planes, measuring less than 1 cm, is consistent with previous studies [18]. This limited space for successful needle insertion further supports the systematic use of ultrasound. The need for preprocedural ultrasound to improve intrathecal access is consistent with prior findings, which report a 74.4% first-attempt success rate (compared to 53.8%) in patients with difficult landmarks, scoliosis, or a history of spine surgery [13]. Similar conclusions have been drawn by other authors for patients with challenging spinal anatomy [19]. Comparable studies in obstetric patients have shown that total procedure time does not significantly differ between ultrasound and palpation groups, as the longer identification phase in the ultrasound group is offset by a shorter procedural time. These studies report a first-attempt success rate of 85.7% [20], and the use of ultrasound has already been proposed as a standard of care in systematic reviews and meta-analyses [21].

The use of 3D imaging improves our understanding of the anatomical structures involved in spinal anesthesia [10], and it helps here to visualize the relationship between the proximal and lateral parts of the transverse and the inferior and superior planes where a perpendicular needle insertion may be successful when a midline approach is not feasible, provided the needle is inserted at the correct pre-estimated angle.

### Limitations of This Study

The deformation of vertebral bodies due to the degenerative process in the elderly (most of the donors were above 80 years old) may affect the quantification of the height of the range of viable perpendicular paths and may alter the percentage of patients without them. Although small, this range likely reflects the narrow margin for feasible planes in elderly surgical patients.

Visualizing the estimated optimal angle in the sterile clinical setting remains an area requiring optimization. To address this, some authors have demonstrated the use of protractors alongside the ultrasound probe to determine the optimal angle when targeting the longest observable ligamentum flavum–dura mater complex [22].

An additional challenge involves preventing needle deviation within tissues [23], which is critical to minimize risks such as bone contact, needle deformation, cerebrospinal fluid leakage, or cauda equina nerve root injury. Although the use of cadaveric specimens, which differ in tissue consistency from living patients, may influence such angulations, the transverse relationship with the vertebral body remains unaffected. Therefore, the anatomical correlations described here are relevant for determining the optimal level for perpendicular needle insertions, regardless of patient age.

Ongoing clinical studies aim to confirm the potential improvements in paramedian neuraxial anesthesia [24,25].

## 5. Conclusions

A high proportion of elderly spines lack a viable median pathway for needle insertion during spinal anesthesia, and the available range for perpendicular paramedian approaches is less than 1 cm in height. The superior aspect of the transverse process serves as a reliable anatomical landmark for stimating dural sac depth, calculate optimal paramedian angles, and selecting the appropriate site for paramedian needle insertion.

## Figures and Tables

**Table 1:** The quantification of the height (in mm) of viable paths for perpendicular needle insertion at the L3–L4 and L4–L5 intervertebral levels. If no midline path is viable, a perpendicular needle insertion should not be attempted.

Case	L4–L5 mm Sup-Inf	Viable Median Path	L3–L4 mm Sup-Inf	Viable Median Path
1	8.5	yes	9.9	yes
2	6.9	yes	8.6	yes
3	3.5	no	6.3	no
4	12.5	yes	6.7	yes
5	9.2	yes	9.3	yes
6	7.7	yes	10.0	yes
7	9.9	yes	8.1	yes
8	13.0	yes	9.4	yes
9	10.2	yes	6.4	no
10	5.6	no	4.2	no
Mean	8.7	80%	7.9	70%
±SD	2.9		1.9	

**Figure A1:**
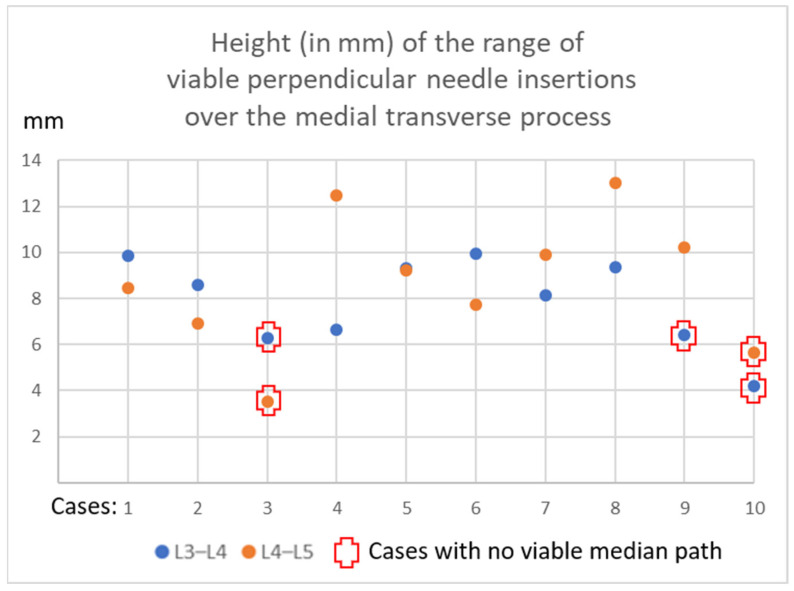
Height (in mm) of the range of viable perpendicular needle insertions over the medial transverse process.

## Data Availability

The raw data supporting the conclusions of this article will be made available by the authors on request.

## Supplementary Materials

The following supporting information can be downloaded at https://www.mdpi.com/article/10.3390/neurosci6040119/s1.
