# Supplementary figures and images for: The Transverse Process as a Landmark for Estimating Dural Sac Depth and Feasible Planes for Optimized Paramedian Needle Insertions
Source: NeuroSci. 2025 Nov 21;6(4):119. doi: 10.3390/neurosci6040119 (PMC12641910; doi:10.3390/neurosci6040119)

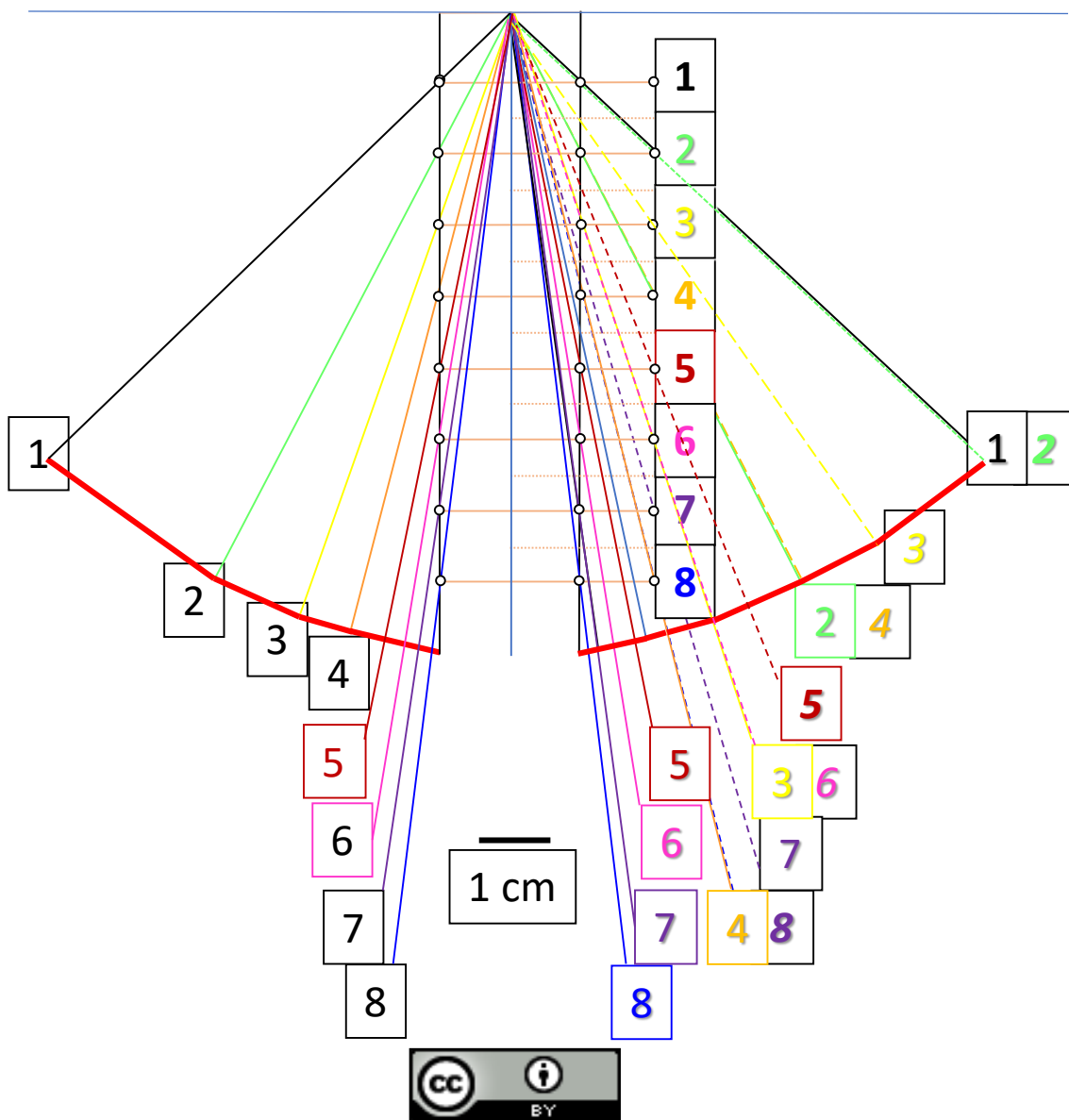

Supplement: Supplementary file 1 [file neurosci-06-00119-s001.zip › neurosci-3944583-supplementary.pdf]
